# Multi-Response Kinetic Modeling of the Maillard Reaction in Milk During Heating at Ultra-High-Temperature Range

**DOI:** 10.1021/acs.jafc.5c14296

**Published:** 2026-05-14

**Authors:** Aytül Hamzalıoǧlu, Işıl Aktaǧ, Vural Gökmen

**Affiliations:** † Food Quality and Safety (FoQuS) Research Group, Department of Food Engineering, 37515 Hacettepe University, 06800 Beytepe, Ankara, Turkey; ‡ Department of Culinary Arts and Gastronomy, 187478 Munzur University, 62000 Aktuluk, Tunceli, Turkey

**Keywords:** Ultrahigh-temperature treatment, milk, Maillard reaction, multiresponse kinetic modeling, advanced glycation end-products\

## Abstract

This study developed first a comprehensive multiresponse kinetic model to elucidate the mechanism of the Maillard reaction in milk during ultrahigh-temperature treatment. The model incorporates the alterations in precursors (sugars, amino acids), intermediates (α-dicarbonyl and Amadori compounds) and products (*N*-ε-carboxymethyllysine, *N*-ε-carboxyethyllysine) that occur in milk during heating between 110 and 140 °C. Kinetically important reactions steps were identified by multiresponse kinetic modeling. Results indicated that *N*-ε-carboxymethyllysine and *N*-ε-carboxyethyllysine were formed predominantly by the oxidation of lactulosyllysine. Among the intermediates, the formation of 1-deoxyglucosone and glucosone, and methylglyoxal from 1-deoxyglucosone and glyoxal from glucosone, were determined to be the kinetically important steps. The rate-limiting step was identified as lactulosyllysine formation, while methylglyoxal generation occurred most rapidly across all heating temperatures. In this study, the multiresponse kinetic modeling approach was applied to milk, offering a comprehensive understanding of the critical pathways leading to the formation of α-dicarbonyl compounds and advanced glycation end-products.

## Graphical abstract

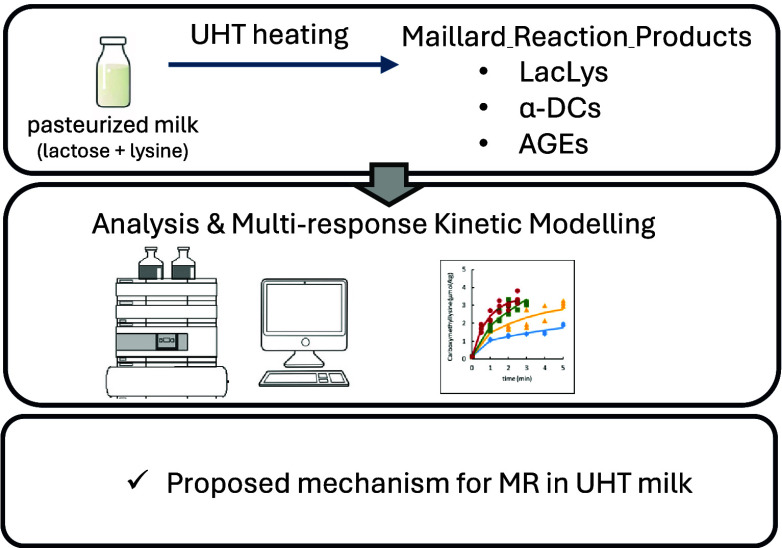

## 1 Introduction

Milk is one of the unique nutritious foods, thanks to its balanced protein (3.2%–3.5%), carbohydrates (4.6%–5.0%), lipids (3.5%–5.0%), and water (87%) content.^1^ In addition to this, free amino acids range from 0.35 μg/mL (tyrosine) to 49.20 μg/mL (glutamic acid) in cow milk, while free lysine content was reported to be 7.81 μg/mL.^2^ However, its composition makes it a favorable medium for microorganisms and thus limits the shelf life. Consequently, to extend its shelf life, high-temperature processing is commonly applied to milk. Direct or indirect ultrahigh-temperature (UHT) processing involves the heating of milk at a temperature higher than 135 °C and followed by aseptic packaging, so that sterility can be guaranteed during storage at room temperature.^3^ According to European Union (EU) Dairy Chemists’ Group, processing at 135 °C should have a minimum sterilization value of *F*_0_ = 3 min as hygiene prescription; however, the upper limit in this process has been prescribed as heating at 145 °C for 10 s.^4^ Such thermal conditions do not only lead to inhibition of microorganisms but also some chemical changes in milk composition.^5^ Alteration of substantial components as well as formation of new compounds occur simultaneously during the UHT treatment of milk.

The Maillard reaction (MR), the reaction between a carbonyl compound and an amine, is the responsible for major changes that occur in milk during UHT treatment.^6^ In the case of milk, MR occurs between reducing sugar, lactose, with ε-amino group of lysine residues of milk proteins.^7^ This first step results in formation of a Schiff base and is followed by the formation of early MR product, Amadori product, such as ε-*N*-deoxylactulosyl-lysine (lactulosyllysine).^8^ During heating, Amadori products degrade to form α-dicarbonyl compounds (α-DCs) in the intermediate stage.^9^ Among α-DCs, 3-deoxyglucosone (3-DG), 3-deoxygalactosone (3-DGal), 3,4-dideoxyglucosone-3-ene (3,4-DGE), 3-deoxypentosone (3-DP), methylglyoxal (MGO), and glyoxal (GO) are the ones mostly formed in milk.^10^ α-DCs are highly reactive intermediates that play a dual role in the MR, contributing to both desirable and undesirable outcomes in milk processing. These compounds readily react with amino and thiol groups of proteins and peptides, leading to the formation of advanced glycation end-products (AGEs) and influencing flavor, color, and nutritional quality.^11,12^ In addition to their formation through the MR, α-DCs can also be generated via sugar degradation pathways, particularly under high heat conditions.^13^ Additionally, in the advanced stage, it was evidenced that *N*-ε-carboxymethyllysine (CML) and *N*-ε-carboxyethyllysine (CEL), which are called as AGEs, are formed in milk due to oxidation of Amadori compounds.^14–16^ These AGEs might be formed in foods through autoxidative glycosylation pathway, as well as Namiki pathway.^17,18^

One of the most significant nutritional consequences of the MR during UHT of milk is the lysine loss. Lysine residues in milk are blocked by carbonyl compounds and thus becomes no longer available for digestion.^19^ The nutritional value of proteins can also be compromised due to alterations in their structure and function, affecting the overall amino acid profile of the milk. Beyond nutritional aspects, α-DCs formed during thermal processing have been discussed in the literature with respect to potential biological effects. Although the relevance of their dietary intake for human health remains a subject of ongoing and controversial debate, the intake of α-DCs from foods and/or their occurrence in vivo may pose health risks by contributing to dicarbonyl stress.^20^ It is also known that they—in particular, 3-DG, MGO, and GO—are prone to react with DNA and tissue proteins in the body.^21,22^ α-DCs also cause the formation of AGEs that can be considered as potentially harmful compounds. Although the causal relationship between dietary AGE intake and disease development in humans remains unclear, the observational and mechanistic studies reported that AGEs are associated with the various degenerative diseases including Alzheimer disease, diabetes, cardiovascular, metabolic and renal diseases.^21^ Moreover, in recent studies, it has been stated that the accumulation of CML in the body is related with the development of some types of cancer such as breast cancer and pancreatic cancer.^23,24^

The chemical changes caused by the MR in milk will increase in parallel with the increasing thermal load in heat treatments applied at high temperatures. Labropoulos, Lopez, and Baker^25^ reported that more than 80% of the chemical changes in milk treated with indirect UHT were due to the time spent in the heating process and less than 2% were due to the time spent in the cooling phase.^25^ Accordingly, minimizing the exposure time of milk to high temperatures is an effective strategy to reduce the chemical changes that occur during thermal processing. Considering the composition of milk, a high amount of reducing sugars, available free amino acids, and neutral pH, it is essential to investigate the formation of MR products during UHT processing. Research into MR products is expanding, with growing evidence highlighting both their potential health risks and their benefits. At the same time, the consumption of UHT-treated milk has continued to increase. Therefore, it has become increasingly important to assess the associated nutritional losses in UHT-treated milk. It is crucial to consider not only vitamin degradation and protein denaturation but also lysine loss and the formation of AGEs, which may have negative impacts on human health. The existing literature provides kinetic data on various MR products, including lactulose, HMF, and furosine formation.^26^ In addition to these, degradation of lactose and its reactions with protein-bound lysine leading to formation of different carbonyl compounds were confirmed in milks heated at high temperatures as well as in milk-like heated systems.^27,28^

However, many earlier studies, employing single-response kinetic approaches, lack comprehensive information regarding the reaction mechanisms. To make better quantitation, it is essential to assess the reaction mechanism and predominant pathways. The MR and caramelization proceed together, consisting of parallel and consecutive reactions, one influencing the other; therefore, discrimination of some common intermediates is challenging in a complex food matrix. Multiresponse kinetic modeling is a powerful tool for unraveling such complex reaction mechanisms, providing deeper understanding on how reactants and products are quantitatively linked. This study aimed to develop a multiresponse kinetic model to better understand the complex interactions and transformations of reactants and products during UHT processing. Certain intermediates formed through MR and lactose degradation were specifically investigated to understand their critical roles. By integrating kinetic parameters, this model offers a calculation tool capable of facilitating industry monitoring and optimization of thermal processes, particularly for potential future regulatory limits on chemical hazards.

## 2 Materials and Methods

### 2.1 Chemicals and Consumables

Lactose was obtained from Sigma–Aldrich (Diesenhofen, Germany). Lysine (>98%) was purchased from Merck Co. (Darmstadt, Germany). 3-DG (75%), glucosone (G), DA, MGO, GO, quinoxaline (99%), 2-methylquinoxaline (97%), MGO (40%), 2,3-dimethylquinoxaline (97%), 5-methylquinoxaline (98%), *o*-phenylenediamine (98%), diethylenetriaminepentaacetic acid (DETAPAC) (98%), ammonium formate (97%), l-theanine, and sodium borohydride powder (≥98%) were purchased from Sigma–Aldrich (Steinheim, Germany). Furosine standard was purchased from Neosystem Laboratoire (Strasbourg, France). Formic acid (98%), methanol, and acetonitrile were obtained from JT Baker (Deventer, Holland). Potassium hexacyanoferrate, zinc sulfate, disodium hydrogen phosphate anhydrous, sodium dihydrogen phosphate dihydrate, sodium hydroxide, boric acid, hydrochloric acid (37%) were purchased from Merck (Darmstadt, Germany). Syringe filters (nylon, 0.45 μm) and Oasis HLB cartridge were supplied by Waters (Milford, MA). Ultrapure water was used throughout the experiments (Milli Q-System, Millipore, Milford, MA).

### 2.2 Lab-Scale UHT Processing of Milk

Industrial UHT processing involves pasteurization of milk prior to UHT. For this reason, pasteurized whole milk purchased from local market was used to simulate this process in the laboratory. Lab-scale heat treatments of milk were performed using a heating metal block at different temperatures and times (ranging from 0.5 to 5.0 min depending on temperature) to determine the changes in the concentrations of precursors, intermediates and products related to the MR. The milk was heated at 110 °C for 1, 2, 3, 4, and 5 min, 120 °C for 1, 2, 3, 4, and 5 min, 130 °C for 1, 1.5, 2, 2.5, and 3 min, and 140 °C for 0.5, 1, 1.5, 2, and 2.5 min. The data was utilized to construct a multiresponse kinetic model for the MR in UHT-treated milk, enabling the estimation of the kinetic rate constants.

To minimize the time lag for the temperature of the milk sample to increase to the set value (<5 s), 1 mL of milk was transferred to preheated glass tubes. To ensure accurate temperature delivery, the temperature rise was continuously monitored with embedded thermocouples. A temperature range between 110 and 140 °C has been used to cover temperatures that may overlap with industrial processes. In addition, long-duration (up to 5 min) heat treatment data include conditions not encountered in industry, allowing for an understanding of the limits. Subsequently, the glass tubes were immersed in an ice bath to stop the MR in the samples. Heat-treated samples were subsequently stored at −80 °C prior to conducting chemical analyses. All heat treatments were performed in triplicate to ensure the reproducibility and reliability of the results.

### 2.3 Extraction of Milk Samples

All UHT-treated milk samples were frozen at −80 °C and freeze-dried. Freeze-dried milk samples were stored at −20 °C prior to analysis. A quantity of 250 mg of dried powder was triple extracted with water (1.25–0.625–0.625 mL) by vortexing for 3 min. Supernatant was removed from retentate and combined in a new tube. For lactose analysis, Carrez clarification was done whereas acetonitrile clarification was performed in the analysis of DCs and free lysine. In Carrez clarification, combined extract was mixed with 50 μL of Carrez I and 50 μL of Carrez II solutions. In acetonitrile clarification, 500 μL of combined extract was mixed with 500 μL of acetonitrile. The mixture was centrifuged at 10 000*g* for 5 min.

### 2.4 Analysis of Lactose

Sugars were determined using an analytical method described previously with minor modifications.^29^ A quantity of 1 mL of extract was mixed with 50 μL Carrez I and 50 μL Carrez II for Carrez clarification. After stirring, the mixture was centrifuged at 8000*g* for 3 min, and then, supernatant was filtered through a preconditioned Oasis HLB cartridge. The cartridges were preconditioned by passing 1 mL methanol and 1 mL water subsequently. The first 8 drops of the sample eluent were discarded and the rest was collected into an autosampler vial.

Analysis of lactose was performed on Agilent 1200 HPLC system consisting of a quaternary pump, an autosampler, a column oven, and a refractive index detector. An isocratic elution with a mobile phase consisting of 0.1% sulfuric acid in water (v/v) at a flow rate of 1.0 mL/min was used. A quantity of 5 μL of sample was injected into a Shodex RSpak KC-811 column (300 mm × 8 mm i.d.) (Tokyo, Japan) conditioned to 40 °C. Quantitation of lactose was according to the external calibration curves built between the concentrations of 0.005%–1%.

### 2.5 Analysis of Free and Protein-Bound Lysine

For the analysis of free lysine, milk samples were extracted as described above and the coextracted colloids were precipitated by mixing with acetonitrile. The extract was filtered through 0.45 μm syringe filter and taken into a vial.

For the analysis of bound lysine, acid hydrolysis procedure was applied to samples using an analytical method described previously.^30^ Twenty-five milligrams of freeze-dried milk powder were weighed into a glass tube and 2.5 mL of 8 N HCl was added onto it. After the headspace of the tubes was flushed with nitrogen gas, screw caps were firmly closed. Hydrolysis was carried out in an oven at 110 °C for 23 h and the hydrolysates were subsequently filtered through filter paper.

A quantity of 100 μL of the acid hydrolysates was placed into a glass tube and purged with nitrogen gas. Then, the residue was dissolved in 1 mL of the mixture of acetonitrile:water (50:50 v/v) and passed through an Oasis HLB cartridge preconditioned with 1 mL of methanol and 1 mL of water. Drops, except for the first eight, were placed into a vial.

Lysine content of both extracts and acid hydrolysates were analyzed by a Agilent Ultivo Triple Quadrupole MS system coupled to Agilent 1260 HPLC in positive mode. Chromatographic separation was performed in Sequant-ZIC-HILIC (5 μm, 4.6 × 250 mm) column at 30 °C using a gradient mixture of (A) 0.1% of formic acid in water and (B) 0.1% of formic acid in acetonitrile as the mobile phase at a flow rate of 0.5 mL/min. The gradient mixture started from 20% A conditioning for 3 min and increased to 60% A in 2 min. After it remained at 60% A for 3 min, it decreased to 20% A in 1 min and remained for 3 min. Chromatographic run was completed in 12 min. The electrospray source had the following settings: capillary voltage of 1.5 kV; nozzle voltage of 500 V; source temperature at 300 °C; sheath gas temperature at 375 °C; desolvation gas (nitrogen) flow of 10 L/min. Sheath gas flow was set to 12 L/min and nebulizer pressure was 40 psi. Theanine was used as an internal standard, and the lysine was identified by multiple reaction monitoring (MRM) of two channels. Both the precursor and product ions were monitored, and quantitation of the lysine concentration in samples was done by virtue of calibration curve built in the range between 1 and 100 μM (1, 2, 5, 10, 20, 50, and 100 μM).

### 2.6 Analysis of Furosine and Lactulosyllysine

To analyze furosine, acid hydrolysates of milk samples were used. A quantity of 100 μL of the acid hydrolysates were put into a glass tube and purged with N_2_ gas. Then, the residue was dissolved in 1 mL of deionized water, passed through an Oasis HLB cartridge preconditioned with 1 mL methanol and 1 mL water. First 8 drops were discarded, and the rest was placed into a vial. The analysis of furosine was carried out, as described previously elsewhere,^31^ on an Agilent 1200 HPLC (Agilent Tech., Waldbronn, Germany) consisting of a diode array detector, a quaternary pump, a temperature-controlled column oven and automatic sample injection system. For the chromatographic separation, Atlantis HILIC column (250 Å × ∼4.6 mm, 5 μm) was used and conditioned to 40 °C. The mobile phase was 1% formic acid in water, and the flow rate was 1 mL/min. Data acquisition was performed at the detector set to 280 nm. Standard solution of furosine with concentrations ranging between 1 and 10 mg/L was prepared, and furosine concentrations of milk samples were calculated by means of creating a calibration curve. According to Brands and van Boekel,^32^ the concentration of Amadori product, LacLys, was obtained from the furosine concentrations of milk samples by multiplying with a conversion factor of 3.1.

### 2.7 Analysis of α-DCs

Analysis of α-DCs was performed according to a previously validated analytical method in infant formulas.^33^ After milk samples were extracted as described above, the coextracted colloids were precipitated by mixing with acetonitrile. A quantity of 500 μL of the combined extract was diluted with 500 μL of the mixture of acetonitrile, and centrifuged at 15 000*g* for 5 min.

For derivatization, 500 μL of supernatant was mixed with 150 μL of 0.2% *o*-phenylenediamine solution containing 11 mM diethylenetriaminepentaacetic acid and 150 μL of 0.5 M sodium phosphate buffer (pH 7). The mixture was immediately filtered through 0.45 μm syringe filter into an autosampler vial. It was kept at room temperature, in darkness, for 2 h prior to LC-MS/MS analysis.

α-DCs were analyzed by an Agilent 1260 Infinity II system coupled to a triple quadrupole detector operated in positive electrospray ionization mode. Chromatographic separations were performed on a Zorbax Eclipse XDB-C18 column (4.6 × 150 mm, 5 μm) by using a gradient mixture of 1% formic acid in water (A) and 1% formic acid in methanol (B) at a flow rate of 1 mL/min. The eluent composition starting with 20% B and then linearly increased to 60% in 8 min. Then, it was decreased to the initial conditions (20% B) in 2 min and held for 3 min. The column was at 40 °C and the autosampler was at 10 °C during the analysis. The electrospray source had the following settings: gas temperature, 250 °C; gas flow, 10 L/min; nebulizer, 60 psi; capillary voltage, 1.5 kV; sheath gas temperature, 400 °C; sheath gas flow, 12 L/min; nozzle voltage, 500 V.

α-DCs were identified by selected ion monitoring (SIM) mode, and the SIM ions [M+H^+^] were as follows for the quinoxaline derivatives of G; 251, 3-DG and 1-DG; 235, DA; 159, MGO; 145, GO; 131. A dwell time was set at 90 ms for each. Working solutions of G and 3-DG were derivatized and then the concentrations of G, 3-DG, quinoxaline, and 2-methylquinoxaline were calculated using calibration curves built in the range between 0.1 and 5 mg/L (0.1, 0.5, 1, 2, 5 mg/L). 5-Methylquinoxaline (5-MeQx) was used as an internal standard (0.5 mg/L). Also, the calibration curve of 3-DG was used for semiquantitation of 1-DG derivatives. All working solutions were prepared in the mixture of acetonitrile:water (50:50 v/v). The retention times of G, 1-DG, 3-DG, GO, MGO, DA, and 5-MeQx were 3.0, 3.5, 4.0, 5.6, 6.6, 7.5, and 8.1 min, respectively.

The limits of detection (LOD) and limit of quantitation (LOQ) were determined at a signal-to-noise ratio of 3 and 10, respectively. Repeatability was assessed by analyzing three replicates on three consecutive days. The method was previously developed and fully validated in milk-based infant formula matrices.^33^ Under these conditions, percentage recoveries were 102.4% for 3-deoxyglucosone, 103.6% for glyoxal, and 99.5% for methylglyoxal, demonstrating high analytical accuracy. Moreover, chromatographic separation and retention time confirmation were established using authentic standards, ensuring the selective determination of 3-deoxyglucosone.

### 2.8 Analysis of CML and CEL

The hydrolysis procedure according to the method described by Charissou, Ait-Ameur, and Birlouez-Aragon^34^ was performed with some modifications.^34^ 20 mg of ground freeze-dried milk was weighed in a glass tube and mixed with 100 μL of deionized water. 450 μL of sodium borate buffer (0.2 M prepared and adjusted the pH of 9.2 by mixing properly 0.2 M boric acid and NaOH) and 500 μL of sodium borohydride (1 M prepared in 0.1 M NaOH) were put into it. Then, the tubes were kept at room temperature for 4 h to transform fructosyllysine to hexitol lysine. Thereafter, 2 mL of 8 N HCl was added, and the caps were closed under nitrogen gas. The tubes were hydrolyzed at 110 °C for 24 h. After cooling to room temperature, 20 μL hydrolysate was transferred into a glass tube and evaporated with nitrogen. After adding 1 mL of deionized water to the residue, it was passed through a preconditioned Oasis HLB cartridge. After the first eight drops, the rest eluent was collected in an autosampler vial for LC–MS/MS analysis. Sample was injected into Waters TQD LC–MS/MS system and the chromatographic separation was performed in an Atlantis T3 column (4.6 Å × ∼150 mm, 3 μm) at 40 °C. The MS system was operated in positive ionization mode using the following interface parameters: source temperature, 120 °C; desolvation temperature, 450 °C; collision gas flow, 0.20 mL/min; desolvation gas flow, 900 L/min; capillary voltage, 3 kV; cone voltage, 25 V; and extractor voltage, 2 V. A mobile phase consisting of 0.1% formic acid in water (A):0.1% formic acid in acetonitrile (B) was used at a flow rate of 0.20 mL/min. The gradient mixture started from 100% A remaining for 5 min and decreased to 70% in 1 min. The instrument was run in multiple reaction monitoring (MRM) mode, and acquisition was performed by monitoring a *m*/*z* ratio of 205.1 for CML and 219.0 for CEL. The precursor ions were fragmented and two product ions having *m*/*z* ratios of 84 and 130 for both CML and CEL were monitored. Quantitation was performed by means of a matrix-matched calibration curve. Pasteurized milk heated at 110 °C for 1 min was used as blank matrix. Calibration solutions of CML and CEL were prepared in the blank matrix at concentrations of 0, 1, 2.5, 5.0, 10, and 20 μg/mL. Then, they were subjected to the lengthy extraction procedure that was used for actual samples and analyzed as described above.

### 2.9 Multiresponse Kinetic Modeling

A comprehensive reaction mechanism was built that consisted of major formation pathways of MR products during UHT processing of milk. Intermediates formed through MR were also added to comprehensive reaction model in order to understand the role of these intermediates on CML and CEL formation. Each reaction step was characterized by its rate constants (*k*) as parameters. The reaction network was introduced to the mathematical model by setting up differential equations for each reaction step (Appendix A). Athena Visual Studio software (v.14.2) (AthenaVisual Inc.) was used for numerical integration, and the parameters were estimated by nonlinear regression using the determinant criterion.^35^ The concentrations of reactants and products were expressed as μmol/kg of milk, and individually measured concentrations of the repetitions were used during modeling. Experimentally obtained data were compared to the mathematical model and the steps in the reaction network were criticized by model discrimination. For each heating temperature, parameter estimation was performed separately. The goodness of fit of the models to the experimental data were used to criticize the kinetic models together with the highest posterior density (HPD) intervals of the estimated parameters. Reparametrized Arrhenius equation was used to determinate the temperature dependence of the reaction rate constants by means of activation energies (*E*_a_, kJ/mol). The reparametrized Arrhenius equation used in the model was given below:

$$k=k_{\mathrm{ref}}\;\exp\left[\frac{E_{a}}{R}\left(\frac{1}{R_{\mathrm{ref}}}-\frac{1}{R}\right)\right]$$

where *k*_ref_ is the rate constant for the reference temperature (*T*_ref_ (K)), *E*_a_ is the activation energy (kJ/mol), and *T* is the heating temperature (K). The reference temperature was set to 120 °C (393.15 K). The mass balance of reactants and products are given in the Supporting Information (Figure S1).

## 3 Results and Discussion

### 3.1 Changes in the Concentrations of Reactants, Intermediates, and Products of the Maillard Reaction in Milk during Heating

First, pasteurized milk were heated at 110, 120, 130, and 140 °C for different times to simulate UHT heating, and the changes in the concentrations of reactants (lactose and lysine), products (CML and CEL) and intermediates (LacLys, 3-DG, 1-DG, G, MGO, GO, and DA) were monitored in heated milk samples. As the main reactant of MR in milk, lactose concentration decreased during heating. In pasteurized milk, lactose concentration was reported to range between 43 and 54 mg/g milk.^36^ Accordingly in this study, lactose concentration was found to be 41.77 ± 1.25 mg/g (939 150 μmol/kg dm) in pasteurized milk before UHT heating; however, it decreased to 37.92 ± 0.75 mg/g (836 600 μmol/kg dm) in milk heated at 110 °C for 5 min. As expected, the reduction in lactose became more pronounced as the heating conditions were intense. Elimination of lactose was 9.21% in the milk UHT heated at 110 °C for 5 min, whereas it rose up to 16.10% in the milk heated at 140 °C for 3 min. Despite lactose being the main carbohydrate in milk, monosaccharides such as glucose, and galactose were also monitored, while a significant change in these monosaccharides could not be detected during heating of pasteurized milk. Similarly, glucose and galactose were not detected in cow milk previously by others.^13,37^

The total lysine content of freeze-dried pasteurized milk powder was 10.4 ± 0.3 g/100 g protein (194 670 μmol/kg dm). The lysine is reported as 8.3 g/100 g protein in lyophilized milk powder after acidic digestion.^38^ As a reactant in MR, lysine tended to decrease upon heating, as well. After heating at 110 °C, percentage loss of total lysine reached 15.9% while 27% of total lysine was eliminated after heating at 140 °C for 2.5 min. Lysine, the most reactive amino acid in milk, participates in MR, therefore, monitoring lysine levels is crucial in assessing the progress of MR in milk.

As mentioned previously, MR also leads to the formation of Amadori compounds, specifically lactulosyl-lysine, fructosyl-lysine, and tagatosyl-lysine, within heat-treated milk.^8^ Acid hydrolysis subsequently converts these Amadori compounds to furosine, which has been recognized as an effective indicator of thermal damage in foods.^39^ Furosine levels after acid hydrolysis of pasteurized milk was found to be 1.04 g/kg protein. However, within heat treatment, it dramatically increased by reaching 3.08 g/kg protein in milk heated at 120 °C for 5 min. Previous studies have observed that furosine levels after acid hydrolysis in UHT milk range from 0.35 to 3.10 g/kg of protein.^40,41^ Amadori compounds are primary intermediates of the MR and serve as key precursors for the subsequent formation of α-DCs.

α-DCs serve as the precursors of desirable volatile aroma-active compounds such as furans, furanones, and Strecker aldehydes, in heat-treated dairy products,^42,43^ as well as undesirable products (AGEs) generated during the MR.^11^ 3-DG, 1-DG, DA, GO, MGO, and G were monitored in pasteurized milk as α-DCs. The amount of 3-DG and 1-DG were found to be 0.31 mg/L (17.19 μmol/kg dm) and 0.04 mg/L (2.43 μmol/kg dm) in pasteurized milk, respectively. However, 3-DG increased to 0.91 mg/L (51.17 μmol/kg dm) and 1-DG increased to 0.21 mg/L (11.79 μmol/kg dm) in UHT-treated milk at 110 °C for 5 min. The results match with those observed in our previous study.^44^ 3-DG ranged between 0.22 and 0.4 mg/L while 1-DG ranged between 0.1 and 0.82 in commercial UHT milk.^44^ Similarly, the amounts of MGO and GO were also consistent with the literature.^10^ Zhang et al.^10^ reported that MGO concentrations in skimmed UHT milk ranged from 0.04 to 0.25 mg/L, while GO concentrations ranged from 0 to 0.15 mg/L. In this study, the amount of MGO present in milk was 0.08 mg/L (10.12 μmol/kg dm) after heating at 110 °C for 5 min, while the amount of GO found in milk was 0.1 mg/L (14.07 μmol/kg dm) after heating at 120 °C for 5 min.

CML, or CEL, is used to indicate the glycation of proteins in foods as markers for the advanced stages of MR. The concentrations of CML and CEL in pasteurized milk were determined to be 0.11 mg/kg protein (0.14 μmol/kg dm) and 0.24 mg/kg protein (0.24 μmol/kg dm), respectively. As expected, the concentration of both CML and CEL dramatically increased by the progress of MR under intense heating conditions. The concentration of CML reached 1.51 mg/kg protein while CEL concentration reached 0.72 mg/kg after heating at 110 °C for 5 min. Scheijen, Clevers, Engelen, Dagnelie, Brouns, Stehouwer, and Schalkwijk^45^ reported that CML concentrations varied from 1 to 10.1 mg/kg protein, whereas CEL concentrations were comparably lower varying from 0.2 to 2.6 mg/kg protein in UHT milk.

### 3.2 Kinetic modeling of the Maillard reaction

The mass balances of reactants and products were also calculated in order to indicate the relative proportion of each compound (%) for all milk samples heated at different temperatures for different times (Figure S1 in the Supporting Information). Heating caused a slight decrease in total moles of both reactants and products at all temperatures. The recovery after the end of heating was calculated to be 89.8%, 86.1%, 82.5%, and 80.6% at 110, 120, 130, and 140 °C, respectively. This loss may be attributed to the formation of numerous not quantitated MR products and minor intermediates that are not explicitly included in the kinetic model.

#### 3.2.1 Reaction Network Model

Initially, a comprehensive reaction mechanism was built including formation pathways for CML and CEL in accordance with knowledge based on literature to assess the MR during the heating of pasteurized milk (Figure 1). As stated above, lactose degraded rapidly, while no detectable levels of the reducing sugars glucose or galactose were observed during UHT heating. This absence may indicate that these monosaccharides, if formed via lactose hydrolysis, undergo subsequent reactions faster than they accumulate to detectable levels. Therefore, neglecting the formation of glucose and galactose, the model included the intermediates produced by lactose isomerization to lactulose and the MR, which yield α-dicarbonyl and lactulosyllysine molecules. Reactions like lactose isomerization, Amadori product formation, α-DC formation through Amadori product degradation, CML and CEL formation through reactions of α-DCs with lysine and transformation through lactulosyllysine, and elimination reactions of α-DCs, lactulose, CML, and CEL were all included in the reaction network.

**Figure 1:**
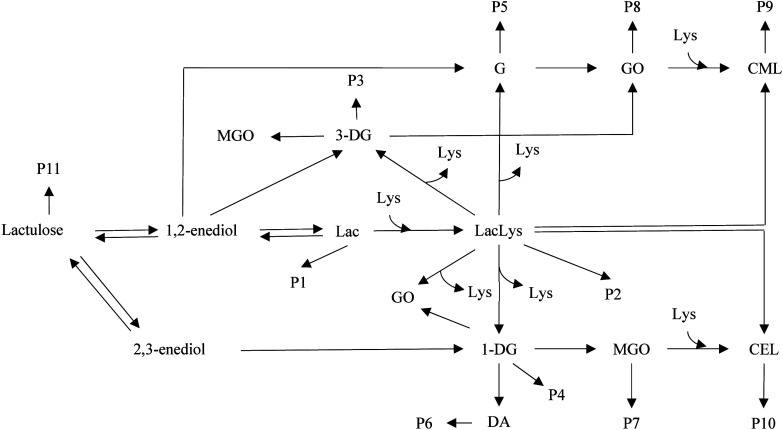
Comprehensive mechanistic model for the Maillard reaction and sugar degradation reactions during heating of pasteurized milk. Lac, lactose; Lys, total (bound and free) lysine; LacLys, lactulosyllysine; 3-DG, 3-deoxyglucosone; 1-DG, 1-deoxyglucosone; G, glucosone; DA, diacetyl; MGO, methylglyoxal; GO, glyoxal; CML, carboxymethyllysine; CEL, carboxyethyllysine; P, products.

The comprehensive model did not provide reliable estimates of the confidence intervals for the rate constants. Therefore, model discrimination simplified the reaction network to increase the precision and reduce the number of unknown parameters.

The Lobry de Bruyn–Alberda van Ekenstein transformation of lactose to lactulose, which can subsequently degrade via a 2,3-enediol anion intermediate, plays a significant role in the initial stages of the MR.^6^ For this purpose, the importance of lactose isomerization to lactulose in heated pasteurized milk was tested by including and excluding the 1,2-enediol intermediate, lactulose, and 2,3-enediol intermediate (not quantitated)—individually and in combination—from the comprehensive reaction network. As reducing sugar, lactose and lactulose could participate in the MR directly or indirectly, and cause the formation of CML or CEL in milk.^46^ However, the results indicated that the reaction rate of this enolization step could not be well-estimated and the model fittings to the experimental data were not well obtained when these steps were included in the mathematical model. Indeed, the degradation reactions via the 1,2-enediol is known to be favored under acidic conditions, whereas the pH of the milk is near neutral. In addition to this, Brands and van Boekel^32^ reported in their study that the lactulose itself was not reactive in the Maillard reaction as a ketose. In another study of Brands and van Boekel,^47^ which is a multiresponse kinetic study on a heated disaccharide–casein model system, 1,2-enediol isomerization of lactose to lactulose was added.^47^ However, the transformation of lactose to lactulose was added to the reaction network as intermediates (INT) in order to obtain the best model fit similar to the study by Brands and van Boekel.^47^ By doing numerous model discriminations, this step was omitted from the kinetic model by comparing the goodness of model fits and parameter uncertainties. Other model practices were also performed to test whether further reaction steps of certain products can be omitted from the kinetic model. For example, the formation of lactose-derived intermediates such as lactosone and 1-deoxylactosone has been reported in the literature;^13^ however, these compounds were not experimentally quantitated in the present study. Therefore, they were included as hypothetical, not quantitated intermediates during model discrimination. However, their inclusion did not improve model performance and led to poorer model fits and increased parameter uncertainty. Similarly, although it has been suggested in the literature that 3-DG formation in lactose-containing systems may proceed via lactose hydrolysis to glucose followed by Maillard reactions,^48^ glucose was not experimentally detected under the applied conditions in this study. Furthermore, the inclusion of glucose as an explicit intermediate during model discrimination resulted in poor model fits and reduced parameter reliability. Therefore, within this kinetic model, the formation of 3-DG is proposed to predominantly proceed via the degradation of Amadori product derived from lactose, rather than through the detectable free glucose intermediate. This assumption is a model-based interpretation aimed at improving the robustness and identifiability of the model, and it should be emphasized that an experimental verification of 3-DG formation via the LacLys pathway is still needed. Finally, some intermediate steps were eliminated from the comprehensive reaction network, and an appropriate model was developed in the end (Figure 2). The proposed model consisted of the formation of Amadori product, α-DC formation via Amadori product degradation, elimination reactions of α-DC, degradation of lysine and CML and CEL formation through degradation of Amadori product, or the reaction of bound lysine with α-DC, as described well in the corresponding section. In general, the model demonstrates consistency with the data, as the fits follow the trend for each compound except for some intermediate compounds under certain temperature conditions (Figure 3). In particular, noticeable deviations were observed for 1-DG, which may be partly attributed to its highly reactive nature, resulting in rapid formation and degradation processes.^49^ In addition, modeling within a real food system inherently complicates the achievement of accurate fits for all intermediate compounds simultaneously.

**Figure 2:**
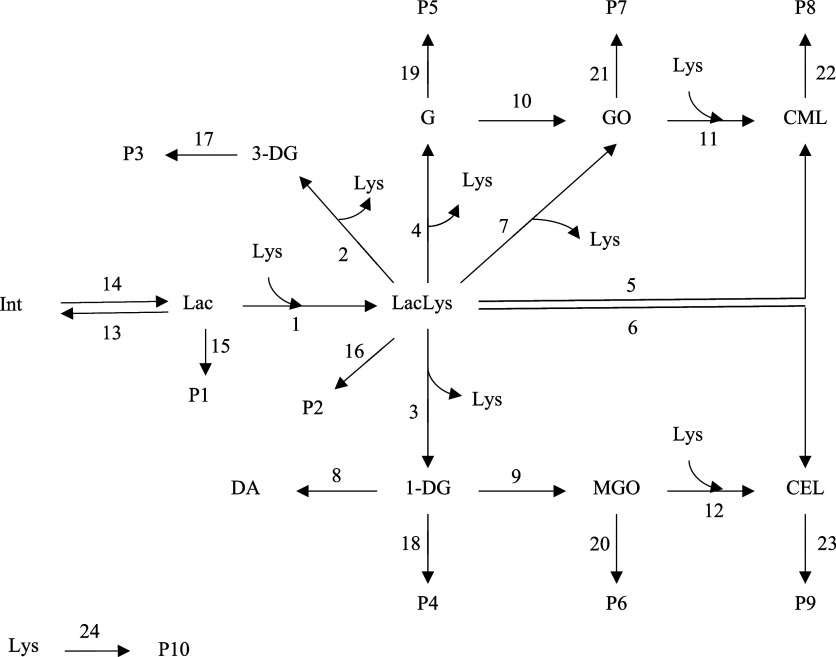
Proposed mechanistic model for the Maillard reaction during heating of pasteurized milk. Lac, lactose; Lys, total (bound and free) lysine; LacLys, lactulosyllysine; 3-DG, 3-deoxyglucosone; 1-DG, 1-deoxyglucosone; G, glucosone; DA, diacetyl; MGO, methylglyoxal; GO, glyoxal; CML, carboxymethyllysine; CEL, carboxyethyllysine; P, products.

**Figure 3:**
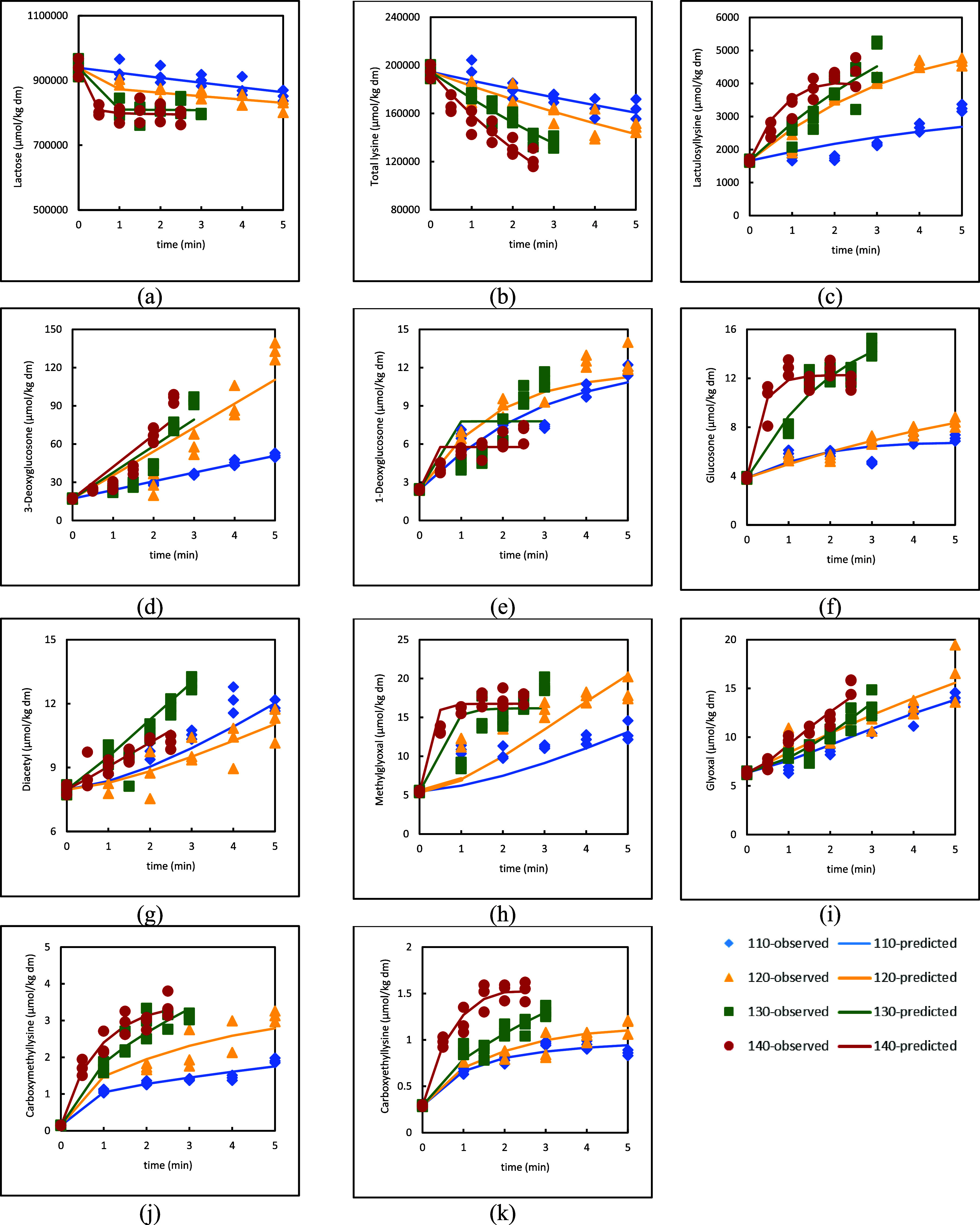
Kinetic model fit (lines) to the experimental data (symbols) of reactants and products during heating of pasteurized milk (a) lactose, (b) total lysine, (c) lactulosyllysine, (d) 3-deoxyglucosone, (e) 1-deoxyglucosone, (f) glucosone, (g) diacetyl, (h) methylglyoxal, (i) glyoxal, (j) carboxymethyllysine, and (k) carboxyethyllysine.

It is well-known that pH of milk typically decreases during heating, which can affect the progress of Maillard reaction. As reported, heating milk at 130 °C for 20 min led to decrease in pH from 6.7 to 5.8. However, it was reported that heating at such high temperatures up to 5 min decrease milk pH only slightly, dropping from 6.7 to 6.6, due to buffering capacity of milk.^50^ In this study, pH change was only 0.1 after heating for 5 min, and such small changes do not fundamentally alter the reaction pathway. Only when substantial amounts of organic acids accumulate—leading to a more pronounced pH drop—can the reaction pathway shift toward acid-favored intermediates. Therefore, formation of organic acids during heating milk were not included in the kinetic model.

Model discrimination revealed the kinetically critical pathways in the reaction network. In this reaction network, numbers indicate the reaction rate constants (*k*) for each elementary reaction step which are given with ±95% Highest Posterior Density (HPD) in Table 1. The differential equations for each elementary reaction step were expressed, and Athena Visual Studio solved these equations simultaneously to generate the rate constants at various UHT heating temperatures. Among 24 rate constants of the reaction steps, 9 rate constants (*k*_3_, *k*_7_, *k*_10_, *k*_13_, *k*_15_, *k*_18_, *k*_19_, *k*_20_, and *k*_23_) were not determined in the ±95% HPD interval at all temperatures. However, these were still included in the model, as their exclusion did not improve the model’s performance. The uncertainty in these steps may have resulted from not quantitated intermediates or trace-level products as well as the complexity of the reaction. The presence of wider HPD intervals might indicate the limitations in parameter identifiability rather than merely large statistical uncertainty. Indeterminate HPD intervals reflect situations in which the uncertainty cannot be reliably estimated, providing information about the model-data relationship. These findings highlight the need for caution when interpreting the affected parameters. To enhance parameter identifiability and reduce redundancy, future studies could consider incorporating more quantifiable intermediates or simplifying the model.

**Table 1:** Estimated Reaction Rate Constants (*k*,) with 95% Highest Posterior Density (HPD) Intervals at Different Temperatures According to the Proposed Kinetic Model in Figure 2 for the Maillard Reaction Products during the Heating of Pasteurized Milk

			110 °C		120 °C		130 °C		140 °C	
Elementary reaction steps		Rate constant unit	*k*	HPD	*k*	HPD	*k*	HPD	*k*	HPD
1	Lac + Lys → LacLys	kg μmol^–1^ min^–1^	2.2 × 10^–9^	±2.6 × 10^–10^	7.5 × 10^–9^	±1.3 × 10^–9^	8.1 × 10^–9^	±1.8 × 10^–9^	2.5 × 10^–8^	±4.5 × 10^–9^
2	LacLys → 3-DG	min^–1^	4.4 × 10^–3^	±1.9 × 10^–3^	5.9 × 10^–3^	±5.7 × 10^–4^	6.8 × 10^–3^	±8.4 × 10^–4^	7.7 × 10^–3^	±8.7 × 10^–4^
3	LacLys → 1-DG	min^–1^	4.1 × 10^–2^	±2.6 × 10^–3^	6.9 × 10^–2^	ind^a^	2.9 × 10^–3^	±2.3 × 10^–4^	1.9 × 10^–1^	±9.2 × 10^–3^
4	LacLys → G	min^–1^	1.9 × 10^–2^	±4.0 × 10^–3^	2.1 × 10^–2^	±1.0 × 10^–3^	2.5 × 10^–2^	±7.1 × 10^–2^	1.5 × 10^–1^	±9.5 × 10^–3^
5	LacLys → CML	min^–1^	1.7 × 10^–4^	±2.6 × 10^–3^	3.6 × 10^–3^	±1.4 × 10^–2^	1.4 × 10^–3^	±2.7 × 10^–3^	1.1 × 10^–3^	±2.3 × 10^–3^
6	LacLys → CEL	min^–1^	0.0	±0.0	1.0 × 10^–3^	±2.8 × 10^–3^	3.3 × 10^–3^	±2.2 × 10^–4^	2.1 × 10^–3^	±1.3 × 10^–3^
7	LacLys → GO	min^–1^	2.5 × 10^–4^	ind^a^	0.0	±0.0	0.0	±0.0	2.0 × 10^–3^	±1.8 × 10^–3^
8	1-DG → DA	min^–1^	1.1 × 10^–1^	±1.2 × 10^–2^	6.5 × 10^–2^	±1.1 × 10^–2^	2.7 × 10^–1^	±2.6 × 10^–2^	2.0 × 10^–1^	±2.8 × 10^–2^
9	1-DG → MGO	min^–1^	10.8	±7.6 × 10^–1^	25.7	±1.2	8.1 × 10^–1^	±9.5 × 10^–2^	123.5	±6.7
10	G → GO	min^–1^	7.4 × 10^–2^	±1.5	3.3 × 10^–1^	±3.9 × 10^–2^	3.5 × 10^–1^	±3.7 × 10^–1^	2.4 × 10^–3^	ind^a^
11	GO + Lys → CML	kg μmol^–1^ min^–1^	0.0	±0.0	8.2 × 10^–7^	±2.5 × 10^–6^	1.9 × 10^–6^	±5.0 × 10^–6^	2.4 × 10^–6^	±4.4 × 10^–6^
12	MGO + Lys → CEL	kg μmol^–1^ min^–1^	8.9 × 10^–7^	±4.5 × 10^–7^	4.1 × 10^–6^	±4.4 × 10^–6^	0.0	±0.0	0.0	±0.0
13	Lac → Int	min^–1^	1.6 × 10^–2^	±3.7 × 10^–3^	4.0 × 10^–1^	ind^a^	9.0 × 10^–1^	ind^a^	8.2 × 10^–1^	±9.0 × 10^–1^
14	Int → Lac	min^–1^	0.0	±0.0	6.4	±3.1	5.7	±0.6	4.8	±5.5
15	Lac → P1	min^–1^	4.1 × 10^–6^	ind^a^	1.2 × 10^–2^	±9.1 × 10^–3^	0.0	±0.0	0.0	±0.0
16	LacLys → P2	min^–1^	0.0	±0.0	3.2 × 10^–2^	±6.0 × 10^–2^	0.0	±0.0	2.9 × 10^–1^	±1.6 × 10^–1^
17	3-DG → P3	min^–1^	9.2 × 10^–2^	±1.4 × 10^–1^	0.0	±0.0	0.0	±0.0	0.0	±0.0
18	1-DG → P4	min^–1^	0.0	±0.0	0.0	±0.0	0.0	±0.0	9.5 × 10^–2^	ind^a^
19	G → P5	min^–1^	6.5	ind^a^	11.4	ind^a^	7.4	±22.4	45.9	ind^a^
20	MGO → P6	min^–1^	8.0	ind^a^	16.1	±1.0	0.0	±0.0	42.8	ind^a^
21	GO → P7	min^–1^	0.0	±0.0	0.0	±0.0	0.0	±0.0	0.0	±0.0
22	CML → P8	min^–1^	2.6	±2.8	6.0	±24.1	2.2	±2.6	2.5	±1.2
23	CEL → P9	min^–1^	2.0	±1.1	14.5	ind^a^	11.4	ind^a^	5.5	±3.5
24	Lys → P10	min^–1^	3.7 × 10^–2^	±6.0 × 10^–3^	5.8 × 10^–2^	±8.0 × 10^–3^	1.2 × 10^–1^	±8.6 × 10^–3^	1.9 × 10^–1^	±1.8 × 10^–2^

a ind = indeterminate, which means a large uncertainty exists in the estimated parameter within the 95% confidence interval.

In addition to the HPD-based assessment, parameter interdependencies were evaluated using the normalized parameter covariance (correlation) matrices obtained from the Athena software (Tables S1 and S2 in the Supporting Information). Table S1 presents the normalized parameter covariance (correlation) matrices obtained at 110, 120, 130, and 140 °C. Most parameter pairs show low to moderate correlations, whereas strong correlations are confined to a limited number of parameters. Many of the strongly correlated reaction steps involve intermediate or transition species that cannot be directly quantitated under the applied experimental conditions. These highly correlated parameters mainly correspond to closely related or consecutive reaction steps involving common intermediates. Such correlations are chemically expected and reflect the structure of the reaction network rather than limitations in parameter identifiability.

Strong negative correlations primarily arise from competition between parallel reaction pathways or from consecutive reactions involving the consumption of shared intermediates, where an increase in the rate of one reaction step reduces the flux through another. Table S2 summarizes strongly correlated parameter pairs across the investigated temperature range. Among the conditions studied, 120 °C shows the lowest number of strong correlations, indicating comparatively improved parameter separability, while at the other temperatures the observed correlations reflect increased pathway coupling rather than poor parameter estimation. Overall, parameter estimation remains chemically consistent across all temperatures.

Temperature dependence of elementary reactions during the heating of pasteurized milk was also tested, and the activation energies were calculated according to the reparametrized Arrhenius equation. Accordingly, the activation energies for each reaction step were found to vary between −205.8 kJ/mol and 89.8 kJ/mol (Table 2). This result was in accordance with the results previously published by Van Boekel,^6^ which indicated that chemical reactions mostly have activation energies of about 120 kJ/mol. The calculated negative activation energy values may suggest that these reaction steps proceed without significant energy barriers;^51^ however, they may also indicate that the corresponding parameters could not be reliably estimated from the available data. The wide 95% HPD intervals indeed point to limited parameter identifiability, which may be attributed to the relatively narrow experimental temperature range.

**Table 2:** Activation Energies (*E*_a_) According to the Proposed Kinetic Model in Figure 2 for the MR during the Heating of Pasteurized Milk

Elementary reaction steps		*E*_a_ (kJ/mol)	HPD
1	Lac + Lys → LacLys	52.1	±19.0
2	LacLys → 3-DG	64.1	±19.8
3	LacLys → 1-DG	89.8	ind^a^
4	LacLys → G	75.9	±21.1
5	LacLys → CML	75.4	±17.6
6	LacLys → CEL	61.5	±19.9
7	LacLys → GO	43.8	±34.7
8	1-DG → DA	18.9	±7.9
9	1-DG → MGO	51.0	±20.1
10	G → GO	4.2	±15.7
11	GO + Lys → CML	–22.4	ind^a^
12	MGO + Lys → CEL	32.6	ind^a^
13	Lac → Int	14.0	ind^a^
14	Int → Lac	–15.4	±15.4
15	Lac → P1	–11.3	±42.3
16	LacLys → P2	17.5	ind^a^
17	3-DG^b^ → P3	–205.8	ind^a^
18	1-DG → P4	–12.5	±867.6
19	G → P5	5.4	ind^a^
20	MGO → P6	43.9	±19.7
21	GO → P7	–0.3	ind^a^
22	CML →P8	43.1	±23.1
23	CEL →P9	9.7	±17.7
24	Lys → P10	23.4	±3.7

a ind = indeterminate, which means a large uncertainty in the estimated parameter is within the 95% confidence interval.

b Indicates that 3-DG corresponds.

#### 3.2.2 Formation of Intermediates (Lactulosyllysine and α-DCs)

The MR is initiated by the reaction of a reducing sugar, such as lactose, which is known to be key factor determining the occurrence of the MR in milk with mainly ε-nucleophilic amino group of lysine residues, guanidino side chain of arginine and N terminus moiety of milk proteins.^7,52^ This is subsequently followed by the Amadori rearrangement, leading to the generation of early MR products such as ε-*N*-deoxylactulosyl-lysine (lactulosyllysine).^8^ The concentration of lactulosyllysine (LacLys), a prevalent marker in thermally processed milk, increases as the intensity of heat treatment increases.^53^ In the heated pasteurized milk samples, the rate constants of the formation of LacLys (*k*_1_) were determined as 2.2 × 10^–9^, 7.5 × 10^–9^, 8.1 × 10^–9^, and 2.5 × 10^–8^ kg μmol^–1^ min^–1^ for 110, 120, 130, and 140 °C, respectively. These results clearly indicate that LacLys formation is significantly enhanced at higher temperatures, particularly at 140 °C. In addition, the formation of LacLys represents the rate-limiting step in the production of α-DCs and advanced MR products, CML and CEL, as it is both the slowest elementary step within the initial stages of this reaction network and is associated with a relatively high energy barrier (52.1 kJ/mol) (Table 2). In addition to degradation of lactose, lysine is primarily consumed through the MR, and its degradation rate (*k*_24_) was found to increase with rising heating temperatures. The reaction rate constant of the degradation of Lys was found as 3.7 × 10^–2^, 5.8 × 10^–2^, 1.2 × 10^–1^, and 1.9 × 10^–1^ min^–1^ for 110, 120, 130, and 140 °C, respectively. Contrarily, Liu et al.^54^ reported that lysine degradation rate remains relatively constant with temperatures in the glucose-lysine model system. This discrepancy may be attributed to the differing physical and chemical characteristics of the model system compared to the real food matrix, such as milk.

Degradation of Amadori product results in the formation of CML, CEL, C-6 skeletal α-DCs, such as 3-DG, 1-DG, and G which were also added to the model (Figure 2). The formation of 3-DG and 1-DG is independent from the presence of oxygen, and they are found in various cyclic hemiacetal structures, mostly α-/β-pyranose and furanose, in aqueous solutions.^55^ 3-DG and 1-DG are generated via enolization and dehydration during sugar degradation, while in the MR, their formation proceeds through enolization and dehydration of Amadori products, followed by hydrolysis of the 3-DG imine leading to amino acid regeneration, whereas in the case of 1-DG the amino acid bound at C-1 is eliminated rather than water.^49^ Elimination of a water molecule from the C-3 of 1,2-enediol is analogue to that of 1,2-eneaminol whereas a water elimination from the first carbon of 2,3-enediol intermediate generates 1-DG while it is formed via regeneration of amino compound from 2,3-eneaminol intermediate.^56,57^ On the other hand, G is generated through oxidative pathway during oxidation of 1,2-enediol intermediate and Amadori products follows by the hydrolysis from first carbon of fructosamine and release of amino acid.^49,58^ In addition to these C6-skeletal DCs, it should be noted that 4-deoxyglucosone (4-DG), which has been reported as one of the primary DCs formed from lactose in model casein-lactose systems,^59^ shares the same protonated molecular ion as 3-DG and therefore cannot be distinguished solely based on *m*/*z* values in LC-MS analysis. Despite the literature evidence indicates that 4-DG is formed at substantially lower molar ratios compared to other C6 DCs (2-DG: 3-DG: 4-DG = 100:50:2.5 in fructose model systems) and represents a highly reactive, short-lived intermediate,^60^ there is limited information regarding the ratio of 3-DG to 4-DG in lactose containing models. However, certain studies have reported the simultaneous formation of 3-DG and 4-DG in lactose-based systems. For instance, Zhang et al.^59^ quantified both compounds in a lactose–casein model system, while Nomi et al.^13^ observed the formation of 4-DG, but not 3-DG, in a lactose–lysine model system. Milk simulates an aqueous system, and mechanistic studies indicate that intermediates formed via the 1,4-glycosidic “peeling-off” route undergo rapid downstream reactions in aqueous systems, as water (OH^–^) at neutral pH promotes cleavage of 1,4-dideoxy-type intermediates, thereby limiting their accumulation in high moisture matrices.^61^ Consistently, lactose-specific degradation of 1,4-linked disaccharides yields downstream products such as 3,4-dideoxy-pentosuloses, supporting the transient nature of 4-DG-related intermediates rather than their substantial accumulation.^60,62^ Nevertheless, further kinetic investigations in real food matrices are required to fully elucidate the relative contributions of individual DCs under processing-relevant conditions.

As indicated by the model discrimination discussed above, the formation of 3-DG, 1-DG, and G via the 1,2-enediol or 2,3-enediol pathways was excluded from the comprehensive model (Figure 1). Consequently, in heated pasteurized milk samples, their formation is attributed to the degradation of the Amadori product, as illustrated in the proposed reaction network (Figure 2). Among the C-6 skeletal α-DCs, the formation of 1-DG and G occurred at a faster rate than that of 3-DG, which is primarily generated through LacLys degradation at all temperatures (Table 1). Moreover, the formation of both 1-DG and G increased with rising heating temperatures, whereas the production of 3-DG remained relatively constant. The stable 3-DG concentrations could be the result of both formation and destruction simultaneously, which is typical for an intermediate. The rate constants of 1-DG, G and 3-DG formation at the highest temperature, 140 °C, were obtained as 1.9 × 10^–1^, 1.5 × 10^–1^, and 7.7 × 10^–3^ min^–1^, respectively. These results are consistent with previous findings indicating that the formation pathway of 3-DG is favored under slightly acidic conditions, whereas the milk samples had similar slightly acidic conditions after heating.^47^

Following the formation of C6-skeletal α-DCs, *retro*-aldol reactions, fragmentation, and water elimination reactions lead to the generation of shorter chain α-DCs.^56^ The most common short chain α-DCs in milk products have been stated in the literature as DA, MGO and GO which are included in the proposed model.^44^ In the DA formation pathway, 1-deoxyglucosone undergoes a rearrangement to form diacetylformoin which is later reduced.^63^ Thus, it was only pathway considered for DA formation in the proposed model. Its formation rate (*k*_8_) ranging in 6.5 × 10^–2^ and 2.7 × 10^–1^ min^–1^ showed a slight increase with increased heating temperature of pasteurized milk. It has been reported that diacetyl mainly formed from sugars in acidic or basic media (above pH 7.0) rather than in neutral conditions.^64^ MGO is formed from 1-DG by the cleavage of C3–C4 bond while it is generated also from 3-DG via the same C3–C4 bond cleavage pathway. However, in the proposed model, MGO formation was incorporated only through the 1-DG pathway, as the formation via the 3-DG route was excluded based on model predictions. MGO formation strongly depends on the factors such as temperature and presence of amino compounds.^49^ Temperature has quite significant effect on the formation of MGO that the temperature increase from 100 to 120 °C more than doubled the MGO level.^68^ Indeed, the rate constant (*k*_9_) showed that the reaction rate of MGO formation (123.5 min^–1^) at 140 °C was almost 10 times higher than that of (10.8 min^–1^) at 110 °C (Table 1). In addition, the formation of MGO was identified as the fastest pathway among the intermediate compounds. This finding supports the notion that MGO formation is not influenced by pH or the presence of oxygen, whereas the formation of other intermediates is strongly affected by environmental factors apart from temperature.^49,65–69^ As the simplest α-DC, GO is formed via both oxidative pathway and carbon skeleton cleavage pathway.^49^ Therefore, G, 1-DG and 3-DG have been suggested as the precursors of GO.^65,70,71^ In addition, it has been indicated that GO is also formed from Schiff bases during MR through Namiki pathway.^72,73^ Among these pathways, only the degradation of G and LacLys was identified by the model as kinetically significant for the formation of GO. The formation of GO via G degradation was found to be nearly 100 times faster than that from LacLys degradation at lower temperatures, 110 °C. The reaction rate constants of the formation of GO via G and LacLys were determined as 7.4 × 10^–2^ and 2.5 × 10^–4^ min^–1^, respectively. However, as the heating temperature increased to 140 °C, the formation rates of GO from both pathways became nearly equivalent, as 2.0 × 10^–3^ and 2.4 × 10^–3^ min^–1^. These results suggest that at lower temperatures, glyoxal formation predominantly proceeds through the G pathway.

In further stages of the MR, reactive carbonyl intermediates contribute to flavor and color development, either through Strecker degradation pathways or by undergoing polymerization to form carbohydrate-based melanoidins.^74^ Therefore, degradation steps for 3-DG, 1-DG, G, MGO, and GO were added to the proposed model characterized with “products” (Px) with the exception of DA whose inclusion did not improve the model fit in this case (Figure 2).

In conclusion, the formation of 1-DG and G rather than 3-DG, the formation of MGO from 1-DG, and the formation of GO from G instead of from 3-DG were identified as kinetically important steps during milk heating. The rate-limiting step in the overall pathway is the formation of LacLys, whereas MGO generation represents the fastest step at all heating temperatures, as it is predominantly influenced by temperature rather than factors such as pH or oxygen availability. At lower temperatures, GO formation mainly proceeds through G oxidation, whereas at higher temperatures, the contributions of G and LacLys oxidation become comparable.

#### 3.2.3 Formation of Advanced Glycation Endproducts (CML, CEL)

In the final stage of the MR, the interaction between α-DCs and nucleophilic side chains of amino acids like lysine results in the production of AGEs such as CML and CEL, which serve as indicators of the advanced MR stage.^75^ CML formation in food systems can occur through multiple pathways. Two main mechanisms have been proposed: one involves the reaction between glyoxal and lysine residues,^76^ while the other occurs via the oxidative degradation of LacLys.^77^ According to the proposed kinetic model, the rate constant for CML formation via GO (*k*_11_) was calculated to be zero at 110 °C and was 1000–10 000 times lower than that of the pathway involving LacLys degradation at temperatures between 120 and 140 °C (Table 1). Nevertheless, this GO pathway was not excluded from the model, because the model fits were not well-estimated in that case. On the other hand, CML formation rate from oxidation of LacLys (*k*_5_) was found to increase as the roasting temperature increased, being 1.7 × 10^–4^, 3.6 × 10^–3^, 1.4 × 10^–3^, and 1.1 × 10^–3^ kg μmol^–1^ min^–1^ at 110, 120, 130, and 140 °C, respectively. In view of these findings, it could be stated that CML formation was predominantly from LacLys oxidation. In support, a study by Nguyen et al.^77^ reported that, in an aqueous model system of sugars and casein, CML generation was reported to mainly be formed from MR by Amadori rearrangement product. Similarly, Liu et al.^54^ showed that the oxidative cleavage of Amadori products represented the primary kinetic pathway for CML formation in a glucose–lysine model system.

CEL, a structural homologue of CML, is predominantly formed through the reaction of MGO with lysine residues. While MGO is considered the principal precursor in CEL formation, the contribution of other reactants has not been fully elucidated.^78^ Blidi et al.^79^ hypothesized that CEL can be putatively formed via preliminary isomerization of glucose into fructose, followed by formation and subsequent fragmentation of the Heyns product glucosyllysine, as also reported by Şen and Gökmen.^80^ In a study by Treibmann et al.^16^ CEL levels were found to correlate with the concentration of Heyns rearrangement products, suggesting a mechanistic link between Heyns product and CEL formation. Considering the possible interconversion between Amadori and Heyns products, lactulosyllysine may give rise to Heyns-type intermediates, whose subsequent fragmentation could contribute to CEL formation in line with previous literature reports.^16,79–81^ In the present model, CEL formation via Amadori product (analogue of Heyns product) was also incorporated, and both reaction pathways were evaluated. Therefore, a reaction pathway involving the formation of a Heyns-type product from LacLys followed by subsequent CEL formation was explicitly tested during model development. However, since the Heyns product could not be experimentally quantitated, the inclusion of this pathway resulted in reduced model reliability. Consequently, CEL formation was ultimately incorporated in the model as occurring indirectly from LacLys, without explicitly resolving the Heyns intermediate. The rate constant of CEL from the oxidative degradation of LacLys (*k*_6_) was relatively higher than the reaction of MGO and bound lysine (*k*_12_), whose rate constants were found to be zero at 130 and 140 °C and incredibly lower at 120 °C. The results revealed that CEL formation from the degradation of LacLys was a kinetically important pathway. Similar to our findings, it was shown by some researchers that Amadori product was contributed more than MGO to the formation of CEL in aqueous model systems of sugars and casein.^77^ On the contrary, Liu et al.^54^ reported that CEL can be formed by the reaction of MGO with lysine residues. However, their study did not investigate the potential contribution of the Amadori product to CEL formation. In addition to these, Nguyen, van der Fels-Klerx and van Boekel^77^ reported that in a sugar and casein MR model, both CML and CEL degraded upon heating; however, the identities of their degradation products remained unidentified. Hence, a degradation step was added for CML and CEL, which was characterized with “products” (P8, P9). Incorporation of these steps improved the model’s fit and contributed to a well-described model solution.

The MR is an inherently complex reaction network involving numerous parallel and consecutive pathways, thereby introducing significant uncertainties, particularly in the formation and degradation of reactive intermediate compounds. Modeling such a complex reaction in real food matrices presents challenges due to matrix heterogeneity, interacting components, and varying processing conditions, which may limit the predictive accuracy of kinetic models. Consequently, future research should prioritize the analysis of intermediate compounds, expand the reaction network, and validate the model across a broader spectrum of food matrices and processing conditions. This comprehensive approach will enhance the model’s robustness and applicability. Despite these challenges, this study presents an in-depth kinetic evaluation of the MR in a real food system, milk. The developed model can be adapted to various thermally processed foods with similar compositions, pH levels, and moisture levels. The findings may support the industrial monitoring, the optimization of thermal processes, and the development of mitigation strategies, particularly for potential regulatory limits on chemical hazards in the future.

## Supplementary Material



## Appendix

Differential equations, which are built from the kinetic model given in Figure 2.

$$\frac{d\left[\mathrm{Lac}\mathrm{Lys}\right]}{dt}=k_{1}\left[\mathrm{Lac}\right]\left[\mathrm{Lys}\right]-k_{2}\left[\mathrm{Lac}\mathrm{Lys}\right]-k_{3}\left[\mathrm{Lac}\mathrm{Lys}\right]-k_{4}\left[\mathrm{Lac}\mathrm{Lys}\right]-k_{5}\left[\mathrm{Lac}\mathrm{Lys}\right]-k_{6}\left[\mathrm{Lac}\mathrm{Lys}\right]-k_{7}\left[\mathrm{Lac}\mathrm{Lys}\right]-k_{16}\left[\mathrm{Lac}\mathrm{Lys}\right]$$

$$\frac{d\left[\mathrm{Lac}\right]}{dt}=k_{14}\left[\mathrm{Int}\right]-k_{13}\left[\mathrm{Lac}\right]-k_{15}\left[\mathrm{Lac}\right]-k_{1}\left[\mathrm{Lac}\right]\left[\mathrm{Lys}\right]$$

$$\frac{d\left[G\right]}{dt}=k_{4}\left[\mathrm{Lac}\mathrm{Lys}\right]-k_{10}\left[G\right]-k_{19}\left[G\right]$$

$$\frac{d\left[3\mathrm{DG}\right]}{dt}=k_{2}\left[\mathrm{Lac}\mathrm{Lys}\right]-k_{17}\left[3\mathrm{DG}\right]$$

$$\frac{d\left[\mathrm{GO}\right]}{dt}=k_{10}\left[G\right]+k_{7}\left[\mathrm{Lac}\mathrm{Lys}\right]-k_{11}\left[\mathrm{GO}\right]\left[\mathrm{Lys}\right]-k_{21}\left[\mathrm{GO}\right]$$

$$\frac{d\left[\mathrm{CML}\right]}{dt}=k_{5}\left[\mathrm{Lac}\mathrm{Lys}\right]+k_{11}\left[\mathrm{GO}\right]\left[\mathrm{Lys}\right]-k_{22}\left[\mathrm{CML}\right]$$

$$\frac{d\left[\mathrm{CEL}\right]}{dt}=k_{6}\left[\mathrm{Lac}\mathrm{Lys}\right]+k_{12}\left[\mathrm{MGO}\right]\left[\mathrm{Lys}\right]-k_{23}\left[\mathrm{CEL}\right]$$

$$\frac{d\left[\mathrm{MGO}\right]}{dt}=k_{9}\left[1\mathrm{DG}\right]-k_{12}\left[\mathrm{MGO}\right]\left[\mathrm{Lys}\right]-k_{20}\left[\mathrm{MGO}\right]$$

$$\frac{d\left[1\mathrm{DG}\right]}{dt}=k_{3}\left[\mathrm{Lac}\mathrm{Lys}\right]-k_{9}\left[1\mathrm{DG}\right]-k_{8}\left[1\mathrm{DG}\right]-k_{18}\left[1\mathrm{DG}\right]$$

$$\frac{d\left[\mathrm{DA}A\right]}{dt}=k_{8}\left[1\mathrm{DG}\right]$$

$$\frac{d\left[\mathrm{Int}\right]}{dt}=k_{13}\left[\mathrm{Lac}\right]-k_{14}\left[\mathrm{Int}\right]$$

$$\frac{d\left[\mathrm{Lys}\right]}{dt}=k_{2}\left[\mathrm{Lac}\mathrm{Lys}\right]+k_{3}\left[\mathrm{Lac}\mathrm{Lys}\right]+k_{4}\left[\mathrm{Lac}\mathrm{Lys}\right]+k_{7}\left[\mathrm{Lac}\mathrm{Lys}\right]-k_{1}\left[\mathrm{Lac}\right]\left[\mathrm{Lys}\right]-k_{11}\left[\mathrm{GO}\right]\left[\mathrm{Lys}\right]-k_{12}\left[\mathrm{MGO}\right]\left[\mathrm{Lys}\right]-k_{24}\left[\mathrm{Lys}\right]$$

$$\frac{d\left[P_{1}\right]}{dt}=k_{15}\left[\mathrm{Lac}\right]$$

$$\frac{d\left[P_{2}\right]}{dt}=k_{16}\left[\mathrm{Lac}\mathrm{Lys}\right]$$

$$\frac{d\left[P_{3}\right]}{dt}=k_{17}\left[3\mathrm{DG}\right]$$

$$\frac{d\left[P_{4}\right]}{dt}=k_{18}\left[1\mathrm{DG}\right]$$

$$\frac{d\left[P_{5}\right]}{dt}=k_{19}\left[G\right]$$

$$\frac{d\left[P_{6}\right]}{dt}=k_{20}\left[\mathrm{MGO}\right]$$

$$\frac{d\left[P_{7}\right]}{dt}=k_{21}\left[\mathrm{GO}\right]$$

$$\frac{d\left[P_{8}\right]}{dt}=k_{22}\left[\mathrm{CML}\right]$$

$$\frac{d\left[P_{9}\right]}{dt}=k_{23}\left[\mathrm{CEL}\right]$$

$$\frac{d\left[P_{10}\right]}{dt}=k_{24}\left[\mathrm{Lys}\right]$$

## Notes

The mass balance of reactants, intermediates, and reaction products during heating of pasteurized milk (PDF)

## Abbreviations

UHT: ultrahigh-temperature
MR: Maillard reaction
Lac: Lactose
ε: *N*-deoxylactulosyl-lysine
LacLys: lactulosyllysine
α-DCs: α-dicarbonyl compounds
G: glucosone
3-DG: 3-deoxyglucosone
3-DGal: 3-deoxygalactosone
4-DG: 4-deoxyglucosone
1-DG: 1-deoxyglucosone
3,4-DGE: 3,4-dideoxyglucosone-3-ene, 3-DP, 3-deoxypentosone
DA: diacetyl
MGO: methylglyoxal
GO: glyoxal
AGEs: advanced glycation end-products
CML: *N*-ε-carboxymethyllysine
CEL: *N*-ε-carboxyethyllysine
INT: intermediates
HPD: Highest Posterior Density
Px: products
