# Supplementary material for: Multi-Response Kinetic Modeling of the Maillard Reaction in Milk During Heating at Ultra-High-Temperature Range
Source: J Agric Food Chem. 2026 May 14;74(20):15776–89. doi: 10.1021/acs.jafc.5c14296 (PMC13220311; doi:10.1021/acs.jafc.5c14296)
Supplement: Supplementary file 1 [file jf5c14296_si_001.pdf]

Supporting Information

Multi-Response Kinetic Modeling of the Maillard Reaction in Milk During Heating at Ultra-High-Temperature Range

Aytül Hamzalıoğlu[1], Işıl Aktağ[1,2], Vural Gökmen[1*]

[1]Food Quality and Safety (FoQuS) Research Group, Department of Food Engineering, Hacettepe University, 06800 Beytepe, Ankara, Turkey

[2]Department of Culinary Arts and Gastronomy, Munzur University, Aktuluk Campus, 62000 Tunceli, Turkey

[*]Corresponding Author: Prof. Dr. Vural Gökmen,

[*]E-mail: vgokmen@hacettepe.edu.tr, tel: 90-312-297-7108, fax: 90-312-299-2123

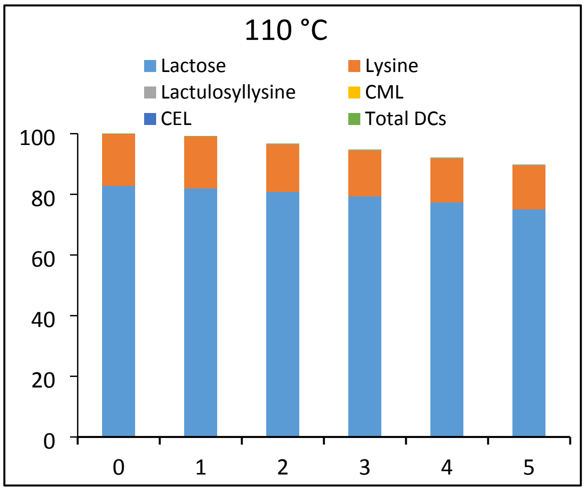

(a)

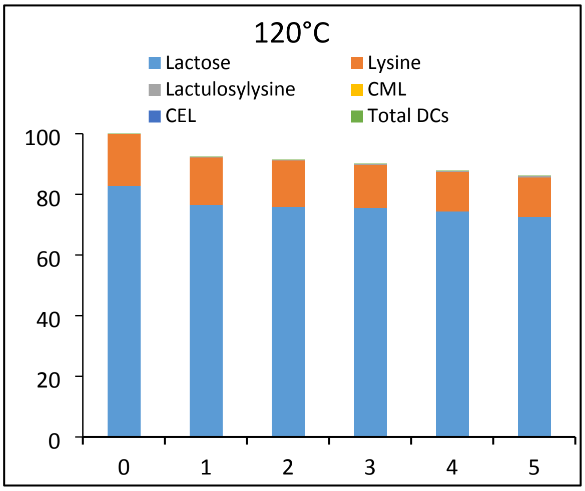

(b)

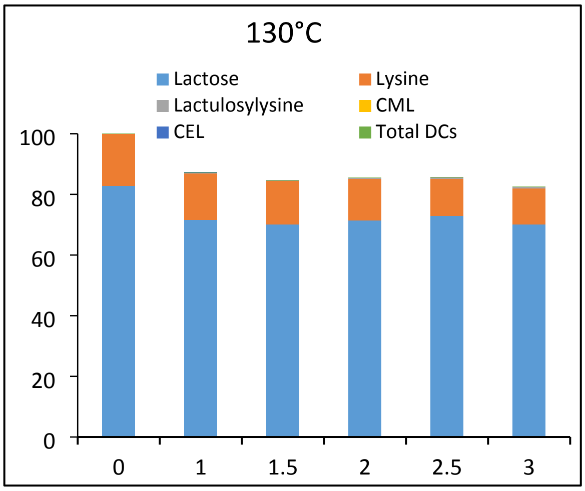

(c)

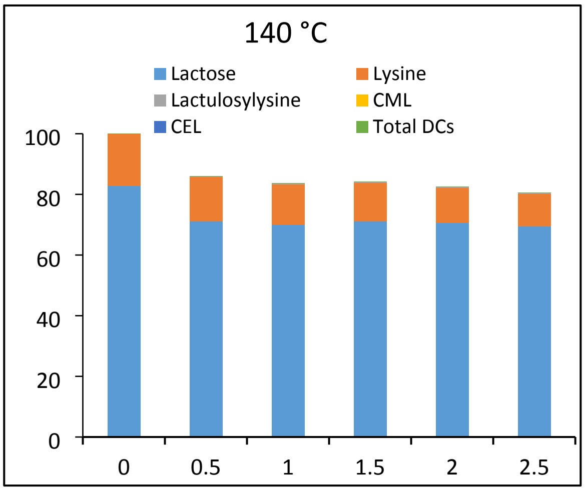

(d)

Figure S1. Mass balance of reactants (lactose, lysine), intermediates (lactulosyllysine, α-dicarbonyl compounds) and reaction products (advanced glycation end-products (AGEs)) during heating of pasteurized milk at (a) 110°C, (b) 120°C, (c) 130°C (d) 140°C

Table S1. Normalized parameter covariance (correlation) matrix obtained from the kinetic modeling results

Normalized parameter covariance matrix at 110 °C

	P(1)	P(2)	P(3)	P(4)	P(5)	P(6)	P(7)	P(8)	P(9)	P(10)	P(11)	P(12)	P(13)	P(14)	P(15)	P(16)	P(17)	P(18)	P(19)	P(20)	P(21)	P(22)	P(23)	P(24)
P*(1)	1																							
P(2)	0.251	1																						
P(3)	-0.75	-0.136	1																					
P(4)	-0.07	-0.032	0.121	1																				
P(5)	-0.12	-0.004	0.053	-0.913	1																			
P(6)	0	0	0	0	0	0																		
P(7)	0	0	0	0	0	0	0																	
P(8)	0.119	0.018	-0.096	-0.021	-0.005	0	0	1																
P(9)	0.11	0.01	0.245	-0.015	0	0	0	0.456	1															
P(10)	0.182	0.012	-0.094	0.938	-0.956	0	0	0.012	0.005	1														
P(11)	0.182	0.012	-0.093	0.943	-0.958	0	0	0.011	0.005	0.999	1													
P(12)	0.405	0.067	-0.259	-0.078	-0.007	0	0	0.036	0.077	0.033	0.032	1												
P(13)	0.054	-0.01	-0.003	-0.006	0.005	0	0	-0.005	-0.002	-0.006	-0.006	-0.008	1											
P(14)	0	0	0	0	0	0	0	0	0	0	0	0	0	0										
P(15)	0	0	0	0	0	0	0	0	0	0	0	0	0	0	0									
P(16)	0	0	0	0	0	0	0	0	0	0	0	0	0	0	0	0								
P(17)	0.344	0.992	-0.213	-0.046	-0.011	0	0	0.03	0.018	0.023	0.023	0.107	-0.009	0	0	0	1							
P(18)	0	0	0	0	0	0	0	0	0	0	0	0	0	0	0	0	0	0						
P(19)	0	0	0	0	0	0	0	0	0	0	0	0	0	0	0	0	0	0	0					
P(20)	0	0	0	0	0	0	0	0	0	0	0	0	0	0	0	0	0	0	0	0				
P(21)	0	0	0	0	0	0	0	0	0	0	0	0	0	0	0	0	0	0	0	0	0			
P(22)	0.244	0.022	-0.14	0.826	-0.725	0	0	0.019	0.011	0.894	0.892	0.069	-0.006	0	0	0	0.039	0	0	0	0	1		
P(23)	0.399	0.066	-0.208	-0.079	-0.002	0	0	0.035	0.116	0.03	0.028	0.995	-0.007	0	0	0	0.105	0	0	0	0	0.067	1	
P(24)	0.076	-0.016	-0.021	0.066	-0.105	0	0	-0.008	-0.027	0.066	0.072	-0.154	-0.001	0	0	0	-0.015	0	0	0	0	-0.01	-0.188	1

Only the lower triangular part of the matrix is shown, as the covariance matrix is symmetric.

**Parameters P(1)–P(24) correspond to the numbered reaction steps shown in Figure 2; each parameter is associated with the corresponding reaction step indicated in the figure.*

Table S1 *(continue)*

Normalized parameter covariance matrix at 120 °C

	P(1)	P(2)	P(3)	P(4)	P(5)	P(6)	P(7)	P(8)	P(9)	P(10)	P(11)	P(12)	P(13)	P(14)	P(15)	P(16)	P(17)	P(18)	P(19)	P(20)	P(21)	P(22)	P(23)	P(24)
P*(1)	1																							
P(2)	-0.25	1																						
P(3)	0	0	0																					
P(4)	-0.31	0.22	0	1																				
P(5)	-0.18	0.019	0	-0.015	1																			
P(6)	0.291	-0.035	0	0.02	-0.078	1																		
P(7)	0	0	0	0	0	0	0																	
P(8)	-0.06	0.005	0	-0.008	0.016	-0.027	0	1																
P(9)	0.306	-0.226	0	-0.496	0.02	-0.026	0	0.129	1															
P(10)	-0.08	0.007	0	-0.159	0.023	-0.038	0	0.008	0.016	1														
P(11)	0	0	0	0	0	0	0	0	0	0	0													
P(12)	-0.3	0.039	0	-0.012	0.078	-0.997	0	0.027	0.018	0.038	0	1												
P(13)	0	0	0	0	0	0	0	0	0	0	0	0	0											
P(14)	-0.19	0.004	0	0	0.008	-0.008	0	0.002	0.001	0.003	0	0.008	0	1										
P(15)	-0.21	0.01	0	0.008	0.012	-0.014	0	0.004	-0.007	0.005	0	0.015	0	0.896	1									
P(16)	0.935	-0.186	0	-0.15	-0.418	0.265	0	-0.066	0.13	-0.09	0	-0.274	0	-0.13	-0.174	1								
P(17)	0	0	0	0	0	0	0	0	0	0	0	0	0	0	0	0	0							
P(18)	0	0	0	0	0	0	0	0	0	0	0	0	0	0	0	0	0	0						
P(19)	0	0	0	0	0	0	0	0	0	0	0	0	0	0	0	0	0	0	0					
P(20)	0.434	-0.196	0	-0.364	-0.038	0.642	0	-0.017	0.377	-0.014	0	-0.632	0	-0.005	-0.015	0.282	0	0	0	1				
P(21)	0	0	0	0	0	0	0	0	0	0	0	0	0	0	0	0	0	0	0	0	0			
P(22)	-0.17	0.016	0	-0.021	1	-0.078	0	0.016	0.026	0.023	0	0.078	0	0.008	0.012	-0.416	0	0	0	-0.034	0	1		
P(23)	0	0	0	0	0	0	0	0	0	0	0	0	0	0	0	0	0	0	0	0	0	0	0	
P(24)	-0.21	0.036	0	0.043	0.026	0.098	0	0.009	-0.041	0.011	0	-0.074	0	0.019	0.029	-0.293	0	0	0	0.033	0	0.025	0	1

**Parameters P(1)–P(24) correspond to the numbered reaction steps shown in Figure 2; each parameter is associated with the corresponding reaction step indicated in the figure.*

Table S1 *(continue)*

Normalized parameter covariance matrix at 130 °C

	P(1)	P(2)	P(3)	P(4)	P(5)	P(6)	P(7)	P(8)	P(9)	P(10)	P(11)	P(12)	P(13)	P(14)	P(15)	P(16)	P(17)	P(18)	P(19)	P(20)	P(21)	P(22)	P(23)	P(24)
P*(1)	1																							
P(2)	-0.32	1																						
P(3)	-0.49	0.243	1																					
P(4)	0.913	-0.199	-0.3	1																				
P(5)	-0.35	0.049	0.075	-0.456	1																			
P(6)	-0.56	0.336	0.509	-0.253	0.025	1																		
P(7)	0	0	0	0	0	0	0																	
P(8)	0	-0.021	-0.089	-0.033	0.022	-0.056	0	1																
P(9)	0.001	-0.017	0.453	-0.027	0.018	-0.045	0	0.213	1															
P(10)	0.303	-0.049	-0.074	0.386	-0.88	-0.04	0	-0.017	-0.014	1														
P(11)	0.342	-0.056	-0.084	0.431	-0.888	-0.045	0	-0.019	-0.015	0.996	1													
P(12)	0	0	0	0	0	0	0	0	0	0	0	0												
P(13)	0	0	0	0	0	0	0	0	0	0	0	0	0											
P(14)	0.005	-0.01	-0.015	0.053	-0.015	-0.022	0	0.002	0.002	0.015	0.017	0	0	1										
P(15)	0	0	0	0	0	0	0	0	0	0	0	0	0	0	0									
P(16)	0	0	0	0	0	0	0	0	0	0	0	0	0	0	0	0								
P(17)	0	0	0	0	0	0	0	0	0	0	0	0	0	0	0	0	0							
P(18)	0	0	0	0	0	0	0	0	0	0	0	0	0	0	0	0	0	0						
P(19)	0.922	-0.207	-0.311	1	-0.443	-0.27	0	-0.032	-0.026	0.371	0.416	0	0	0.053	0	0	0	0	1					
P(20)	0	0	0	0	0	0	0	0	0	0	0	0	0	0	0	0	0	0	0	0				
P(21)	0	0	0	0	0	0	0	0	0	0	0	0	0	0	0	0	0	0	0	0	0			
P(22)	-0.29	0.023	0.036	-0.406	0.958	-0.023	0	0.024	0.019	-0.711	-0.721	0	0	-0.011	0	0	0	0	-0.394	0	0	1		
P(23)	0	0	0	0	0	0	0	0	0	0	0	0	0	0	0	0	0	0	0	0	0	0		
P(24)	-0.03	0.02	0.03	-0.059	0.01	0.054	0	-0.007	-0.006	-0.029	-0.026	0	0	-0.003	0	0	0	0	-0.06	0	0	-0.007		

**Parameters P(1)–P(24) correspond to the numbered reaction steps shown in Figure 2; each parameter is associated with the corresponding reaction step indicated in the figure.*

Table S1 *(continue)*

Normalized parameter covariance matrix at 140 °C	P(1)	P(2)	P(3)	P(4)	P(5)	P(6)	P(7)	P(8)	P(9)	P(10)	P(11)	P(12)	P(13)	P(14)	P(15)	P(16)	P(17)	P(18)	P(19)	P(20)	P(21)	P(22)	P(23)	P(24)
P*(1)	1																							
P(2)	-0.24	1																						
P(3)	-0.38	0.319	1																					
P(4)	-0.28	0.237	0.605	1																				
P(5)	-0.18	0.023	-0.011	-0.009	1																			
P(6)	-0.35	0.043	-0.026	-0.021	0.111	1																		
P(7)	0.11	0.001	0.051	0.038	-0.94	-0.078	1																	
P(8)	-0.07	0.003	-0.017	-0.015	0.023	0.046	-0.017	1																
P(9)	-0.01	0.001	0.163	-0.001	0.003	0.006	-0.002	0.326	1															
P(10)	0	0	0	0	0	0	0	0	0	0														
P(11)	0.136	-0.015	0.011	0.009	-0.944	-0.082	0.997	-0.017	-0.002	0	1													
P(12)	0	0	0	0	0	0	0	0	0	0	0	0												
P(13)	0.383	0.006	0.014	0.011	0.009	0.043	-0.003	0	0	0	-0.003	0	1											
P(14)	0.386	0.005	0.012	0.009	0.008	0.041	-0.002	0	0	0	-0.003	0	0.996	1										
P(15)	0	0	0	0	0	0	0	0	0	0	0	0	0	0	0									
P(16)	0.968	-0.185	-0.212	-0.178	-0.205	-0.401	0.135	-0.082	-0.02	0	0.153	0	0.332	0.341	0	1								
P(17)	0	0	0	0	0	0	0	0	0	0	0	0	0	0	0	0	0							
P(18)	0	0	0	0	0	0	0	0	0	0	0	0	0	0	0	0	0	0						
P(19)	0	0	0	0	0	0	0	0	0	0	0	0	0	0	0	0	0	0	0					
P(20)	0	0	0	0	0	0	0	0	0	0	0	0	0	0	0	0	0	0	0	0				
P(21)	0	0	0	0	0	0	0	0	0	0	0	0	0	0	0	0	0	0	0	0	0			
P(22)	-0.18	0.009	-0.054	-0.041	0.696	0.125	-0.419	0.027	0.003	0	-0.425	0	0.018	0.017	0	-0.202	0	0	0	0	0	1		
P(23)	-0.32	0.018	-0.089	-0.069	0.111	0.996	-0.081	0.047	0.006	0	-0.082	0	0.042	0.041	0	-0.381	0	0	0	0	0	0.128	1	
P(24)	-0.29	0.052	0.084	0.067	0.019	0.078	-0.032	0.014	0.003	0	-0.026	0	-0.071	-0.073	0	-0.389	0	0	0	0	0	-0.042	0.071	1

**Parameters P(1)–P(24) correspond to the numbered reaction steps shown in Figure 2; each parameter is associated with the corresponding reaction step indicated in the figure.*

Table S2. Strongly correlated parameter pairs identified from the normalized covariance matrices at different temperatures.

Parameter i	Parameter j	Correlation (r)
	**110 °C**	
P*(10)	P(11)	0.999
P(12)	P(23)	0.995
P(2)	P(17)	0.992
P(4)	P(10)	0.938
P(4)	P(11)	0.943
P(5)	P(10)	−0.956
P(5)	P(11)	−0.958
P(4)	P(5)	−0.913
	**120 °C**	
P(6)	P(12)	−0.997
P(1)	P(16)	0.935
	**130 °C**	
P(10)	P(11)	0.996
P(5)	P(22)	0.958
P(1)	P(19)	0.922
P(1)	P(4)	0.913
	**140 °C**	
P(6)	P(23)	0.996
P(13)	P(14)	0.996
P(7)	P(11)	0.997
P(1)	P(16)	0.968
P(5)	P(7)	−0.940
P(5)	P(11)	−0.944

**Parameters P(1)–P(24) correspond to the numbered reaction steps shown in Figure 2; each parameter is associated with the corresponding reaction step indicated in the figure.*

Athena Scripts

```
Global Laco,Lyso,LacLyso,DG3o,DG1o,Go,Dao,MGOo,GOo,CMLo,CELo As Real
Laco=939150.00       !initial concentration of lactose uMol/kg
Lyso=194670.00       !initial concentration of total lysine uMol/kg
LacLyso=1661.00      !initial concentration of lactulosyllysine uMol/kg
DG3o=17.10           !initial concentration of 3-deoxyglucosone uMol/kg
DG1o=2.43            !initial concentration of 1-deoxyglucosone uMol/kg
Go=3.85              !initial concentration of glucosone uMol/kg
Dao=7.96             !initial concentration of diacetyl uMol/kg
MGOo=5.45            !initial concentration of methylglyoxal uMol/kg
GOo=6.34             !initial concentration of glyoxal uMol/kg
CMLo=1.83            !initial concentration of carboxymethyllysine uMol/kg
CELo=0.29            !initial concentration of carboxyethyllysine uMol/kg
Global kB(24), Time As Real
Time=5.00            !minute
Global Ea (24) As Real
Global Tb, Temp, Rg As Real
Tb= 120.00           !Reference temp, Celcius
Rg= 8.314            !Universal Gas Constant, J/mol K
@Initial Conditions
U(1)=Laco
U(2)=Lyso
U(3)=LacLyso
U(4)=DG3o
U(5)=DG1o
U(6)=Go
U(7)=Dao
U(8)=MGOo
U(9)=GOo
U(10)=CMLo
U(11)=CELo
```

```
U(12)=0.0 “Int”
U(13)=0.0 “P1”
U(14)=0.0 “P2”
U(15)=0.0 “P3”
U(16)=0.0 “P4”
U(17)=0.0 “P5”
U(18)=0.0 “P6”
U(19)=0.0 “P7”
U(20)=0.0 “P8”
U(21)=0.0 “P9”
U(22)=0.0 “P10”

@Model Equations

Dim a, k(24) As Real

a=1.00-(Tb+273.15)/(Temp+273.15)

k=kB*exp((a*Ea)/Rg*(Tb+273.15))

F(1)=k(1)*U(1)*U(2)-k(2)* U(3)- k(3)*U(3)-k(4)*U(3)-k(5)*U(3)-k(6)*U(3)-k(7)*U(3)-k(16)*U(3)
F(2)=k(14)*U(12)-k(13)* U(1)- k(15)*U(1)-k(1)*U(1)*U(2)
F(3)=k(4)*U(3)-k(10)* U(6)- k(19)*U(6)
F(4)=k(2)*U(3)-k(17)* U(4)
F(5)=k(10)*U(6)+k(7)*U(3)- k(11)*U(9)* U(2)-k(21)*U(9)
F(6)=k(5)*U(3)+k(11)* U(9)*U(2)- k(22)*U(10)
F(7)=k(6)*U(3)+k(12)* U(8)*U(2)- k(23)*U(11)
F(8)=k(9)*U(5)-k(12)* U(8)*U(2)- k(20)*U(8)
F(9)=k(3)*U(3)-k(9)* U(5)- k(8)*U(5)- k(18)*U(5)
F(10)=k(8)*U(5)
F(11)=k(13)*U(1)- k(14)*U(12)
F(12)= k(2)*U(3)+k(3)* U(3)+k(4)*U(3)+k(7)*U(3)-k(1)*U(1)*U(2)-k(11)*U(9)*U(2)-
k(12)*U(8)*U(2)-k(24)*U(2)
F(13)=k(15)*U(1)
F(14)=k(16)*U(3)
F(15)=k(17)*U(4)
F(16)=k(18)*U(5)
F(17)=k(19)*U(6)
F(18)=k(20)*U(8)
F(19)=k(21)*U(9)
F(20)=k(22)*U(10)
F(21)=k(23)*U(11)
F(22)=k(24)*U(2)

@Response Model

Y(1)=U(1)

Y(2)=U(2)
```

Y(3)=U(3)

Y(4)=U(4)

Y(5)=U(5)

Y(6)=U(6)

Y(7)=U(7)

Y(8)=U(8)

Y(9)=U(9)

Y(10)=U(10)

Y(11)=U(11)

@Connect Parameters and Settings

Time=Xu(1)

Temp=Xu(2)

kB(1:24)=Par(1:24)

Ea(1:24)=Par(25:48)

@Solver Options

Neq=22

Npts=48

Tend=Time

VariableNames=Time; Temp; Lactose; TotalLysine; Lactulosyllysine; 3Deoxyglucosone; 1Deoxyglucosone; Glucosone; Diacetyl; Methylglyoxal; Glyoxal; Carboxymethyllysine; Carboxyethyllysine

Headers=Time, minute; Temp, CelciusDegree Lactose, umol/kg; TotalLysine, umol/kg; Lactulosyllysine, umol/kg; 3Deoxyglucosone, umol/kg; 1Deoxyglucosone, umol/kg; Glucosone, umol/kg; Diacetyl, umol/kg; Methylglyoxal, umol/kg; Glyoxal, umol/kg; Carboxymethyllysine, umol/kg; Carboxyethyllysine, umol/kg
